# dGLYAT modulates Gadd45-mediated JNK activation and cell invasion

**DOI:** 10.1186/s13008-022-00080-5

**Published:** 2022-08-06

**Authors:** Meng Xu, Pu Ren, Juhui Tian, Lisha Xiao, Ping Hu, Ping Chen, Wenzhe Li, Lei Xue

**Affiliations:** 1 The First Rehabilitation Hospital of Shanghai, Shanghai Key Laboratory of Signaling and Diseases Research, School of Life Science and Technology, Tongji University, Shanghai, China; 2 Zhuhai Precision Medical Center, Guangdong Provincial Key Laboratory of Tumor Interventional Diagnosis and Treatment, Zhuhai People’s Hospital, Zhuhai Hospital Affiliated with Jinan University, Zhuhai, Guangdong China

**Keywords:** *Drosophila*, Cell invasion, JNK, dGLYAT, Gadd45

## Abstract

**Background:**

Cell invasion is a crucial step of tumor metastasis, finding new regulators of which offers potential drug targets for cancer therapy. Aberrant *GLYAT* expression is associated with human cancers, yet its role in cancer remains unknown. This study aims to understand the function and mechanism of *Drosophila GLYAT* in cell invasion.

**Results:**

We found that dGLYAT regulates Gadd45-mediated JNK pathway activation and cell invasion. Firstly, loss of *dGLYAT* suppressed *scrib* depletion- or Egr overexpression-induced JNK pathway activation and invasive cell migration. Secondary, mRNA-seq analysis identified *Gadd45* as a potential transcriptional target of dGLYAT, as depletion of *dGLYAT* decreased *Gadd45* mRNA level. Finally, *Gadd45* knockdown suppressed *scrib* depletion-induced JNK pathway activation and cell invasion.

**Conclusions:**

These evidences reveal the role of dGLYAT and Gadd45 in JNK-dependent cell invasion, and provide insight for the roles of their human homologs in cancers.

**Supplementary Information:**

The online version contains supplementary material available at 10.1186/s13008-022-00080-5.

## Background

Tumor metastasis, rather than primary tumor formation, is the main cause of fatality in cancer patients [1]. Therefore, identifying additional factors involved in tumor cell invasion and metastasis is of great significance to develop novel strategies for cancer prevention and therapies [2]. Various approaches and model systems have been employed to comprehend the mechanisms underlying cancer metastasis, among which the fruit fly, *Drosophila melanogaster*, has emerged as an excellent model organism to dissect different cancer processes [3]. For example, depletion of cell polarity genes or C-terminal SRC kinase *(Csk)* in *Drosophila* larval wing disc epithelia induces epithelia-mesenchymal transition (EMT)-like cell migration [4].

The Jun N-terminal Kinase (JNK) signaling is evolutionarily conserved from *Drosophila* to human, and plays critical roles in cancer initiation and progression [5]. In *Drosophila*, the JNK pathway consists of a kinase cascade including the JNK kinase kinase such as dTAK1 [6], the JNK kinase hemipterous (Hep) [7], and the sole fly JNK Basket (Bsk) [8]. Upon activation of the kinase cascade by sequential phosphorylation, Bsk phosphorylates and activates downstream transcription factors including Jun and Fos, which form the AP-1 heterodimers [9]. *puckered* (*puc*), a transcriptional target of JNK signaling, encodes a JNK phosphatase that inhibits Bsk activity in a negative feed-back manner [10]. Previous studies in *Drosophila* have found that JNK signaling plays pivotal roles in cell proliferation, migration and apoptosis in normal development, and promotes tumorigenic cell death and invasion in a context dependent manner [11].

Human glycine N-acyltransferases (GLYAT, GLYATL1, GLYATL2, and GLYATL3) promote conjugation of carboxylic acids to glycine and glutamine, which play crucial roles in the detoxification of endogenous and exogenous acyl-CoA. Previous studies suggest potential roles of GLYAT family members in various cancers, for instance, aberrant GLYAT expression has been associated with hepatocellular carcinomas and breast cancer [12], and reduced GLYATL1 expression is correlated with short overall survival in hepatocellular carcinoma patients [13]. On the other hand, data from the Oncomine Platform (https://www.oncomine.org) show that *GLYATL1* mRNA expression is up-regulated in colorectal and prostate cancers, but is down-regulated in kidney and liver cancers. Despite these association, the exact functions of GLYAT family proteins in cancers remain elusive. Our previous work found a *Drosophila* homolog of GLYAT (*dGLYAT*) is required for JNK-mediated cell death [14], yet two critical questions remain unanswered. Whether *dGLYAT* is required for other in vivo functions of JNK pathway, and by which mechanism does *dGLYAT* regulate JNK signaling?

To address the above questions, we took advantage of the well-established *Drosophila* cell invasion model. In this model, knockdown of a cell polarity gene, e.g. *scrib*, *lgl* or *dlg*, along the anterior/posterior (A/P) compartment boundary in 3rd instar larval wing imaginal discs by *ptc*-Gal4, induces JNK-dependent invasive cell migration [15]. To assess whether *dGLYAT* contributes to JNK–mediated cell invasion, we depleted *dGLYAT* by mutation or *RNAi*-mediated knockdown, and found *dGLYAT* is required for JNK-dependent cell invasion. In particular, loss of *dGLYAT* suppresses *scrib* depletion- or Egr overexpression-induced JNK-dependent cell invasion, and impedes *scrib* knockdown-triggered JNK pathway activation. To investigate the mechanism by which dGLYAT regulates JNK signaling, we performed mRNA-seq analysis, and found a significant reduction of *Gadd45* (Growth Arrest and DNA Damage-inducible 45) mRNA level upon *dGLYAT* depletion. Finally, knockdown of *Gadd45* suppresses loss-of-*scrib-*induced JNK activation and cell invasion. Thus, these data provide the first in vivo evidence that dGLYAT modulates JNK-dependent cell invasion through Gadd45.

## Results

### ***dGLYAT*is required for cell polarity disruption-induced cell invasion**

**Fig. 1:**
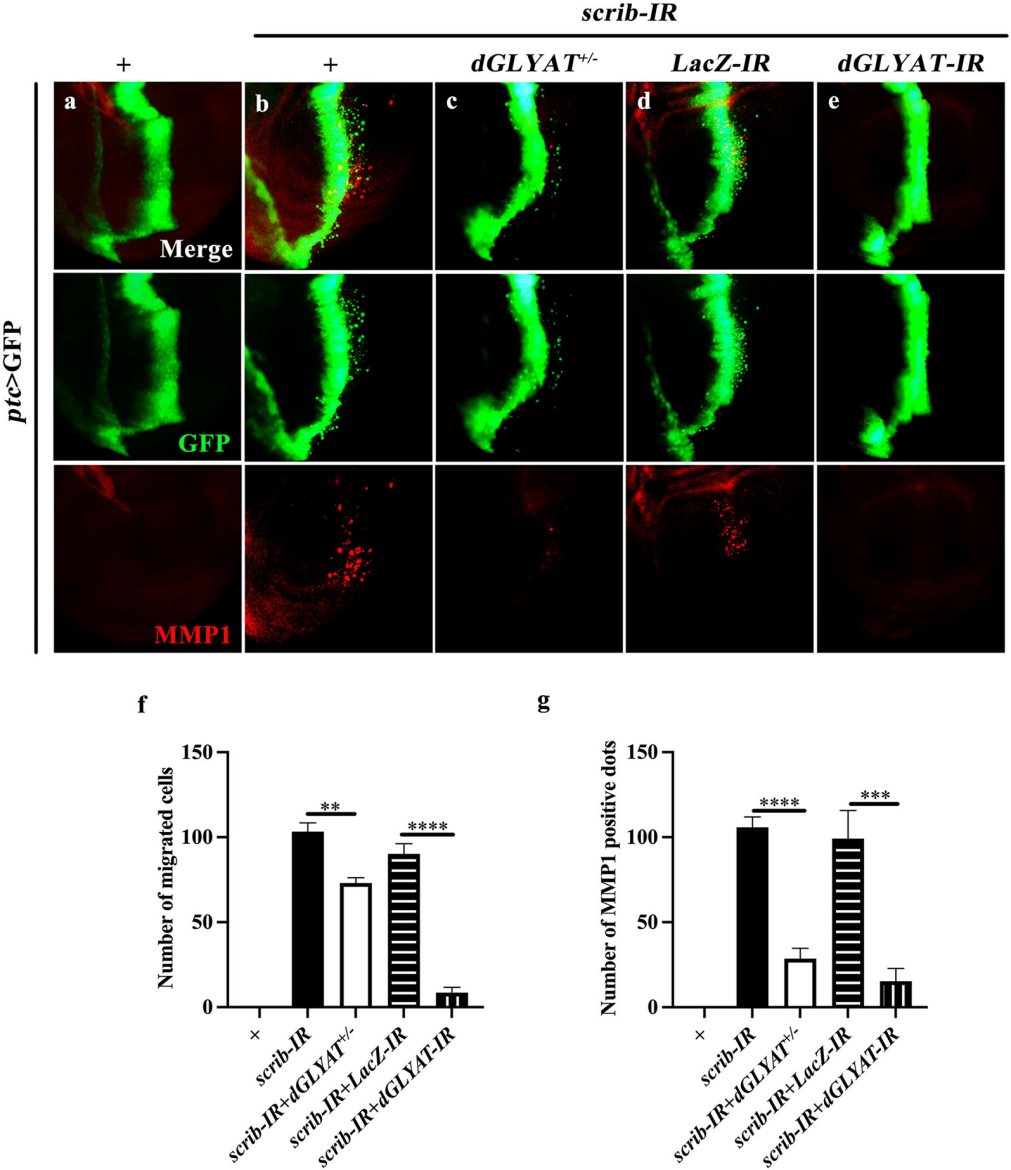
***dGLYAT*** is required for cell polarity disruption-induced cell invasion. Fluorescent micrographs of *Drosophila* third instar larval wing discs are shown (**a**-**e**). Compared with the *ptc* > GFP control (**a**), *ptc* > *scrib-IR* induced cell migration from A/P boundary toward posterior and elevated MMP1 expression (**b**). Both phenotypes were significantly suppressed by heterozygous mutation (**c**) or *RNAi* expression of *dGLYAT* (**e**). *LacZ RNAi* served as a negative control (**d**). **f** Statistic of number of migrated cells is shown (left to right: n = 10, n = 8, n = 5. n = 8, n = 10). **g** Statistic of number of MMP1 positive dots is shown (left to right: n = 10, n = 12, n = 5. n = 5, n = 10). *t*-test was used to compute *P*-values. **P < 0.01, ***P < 0.001, ****P < 0.0001. Detailed genotypes: **a** *ptc*-Gal4 *UAS*-GFP/+, **b** *ptc*-Gal4 *UAS*-GFP *UAS*-*scrib-IR*/+, **c** *ptc*-Gal4 *UAS*-GFP *UAS*-*scrib-IR*/*dGLYAT*^*c02982*^, **d** *ptc*-Gal4 *UAS*-GFP *UAS*-*scrib-IR*/+; *UAS*-*LacZ-IR*/+, **e** *ptc*-Gal4 *UAS*-GFP *UAS*-*scrib-IR*/+; *UAS*-*dGLYAT-IR*/+

It is well known that disrupting cell polarity by depleting *scrib* along the anterior/posterior (A/P) compartment boundary of the wing discs leads to JNK-dependent invasive cell migration [16], in which cells migrate away from the A/P boundary with up-regulated expression of matrix metalloprotease 1 (MMP1) that is able to degrade the basement membrane [17]. Using this well-established in vivo cell invasion model, we examined the role of *dGLYAT* in cell polarity disruption-induced cell invasion. Compared with *ptc* > GFP control (Fig. 1a), knockdown of *scrib* induced extensive cell migration and MMP1 up-regulation (Fig. 1b, quantified in Fig. 1f, g). Both phenotypes were significantly suppressed in heterozygous *dGLYAT*^c02982^ mutants (Fig. 1c), which has a piggyBac insertion into the second exon that deletes the critical Gcn5-related N-acetyltransferases (GNAT) domain [14]. To corroborate this result, we depleted *dGLYAT* by RNAi-mediated knockdown (Additional file 2: Figure S1), and confirmed that expression of a *dGLYAT-IR* dramatically inhibited *scrib* depletion-induced cell migration and MMP1 elevation, compared with a *LacZ* RNAi as a negative control (Fig. 1d-g).

**Fig. 2:**
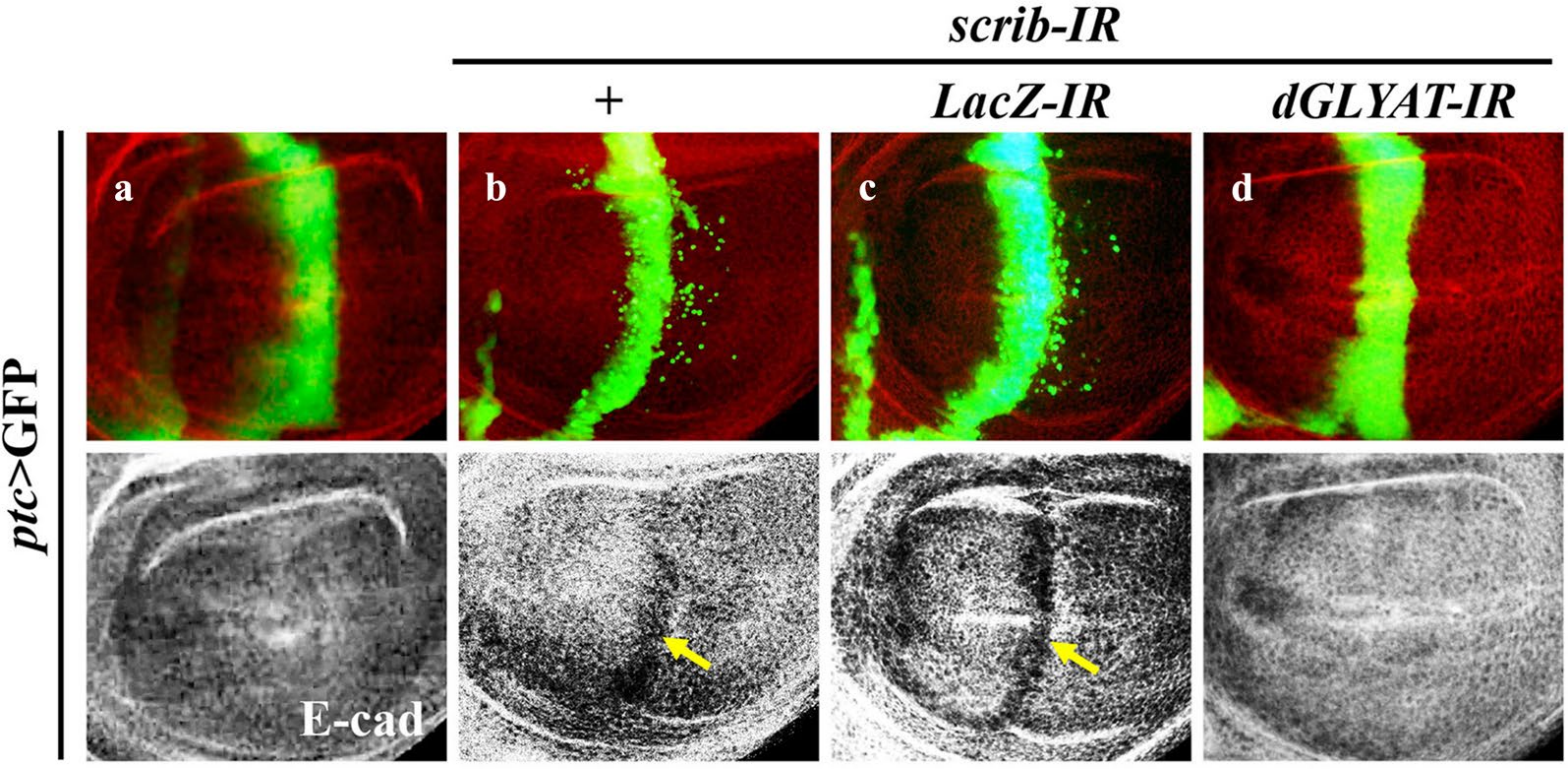
***dGLYAT*** is necessary for cell polarity disruption-induced EMT-like phenotype. Fluorescent micrographs of third instar larval wing discs are shown (**a**-**d**). Compared with the *ptc* > GFP control (**a**), *ptc* > *scrib-IR* induced cell migration and reduced E-cadherin expression in the A/P boundary (**b**, arrow), which were suppressed by expressing *dGLYAT* RNAi (**d**), but not *LacZ RNAi* (**c**). Detailed genotypes: **a** *ptc*-Gal4 *UAS*-GFP/+, **b** *ptc*-Gal4 *UAS*-GFP *UAS*-*scrib-IR*/+, **c** *ptc*-Gal4 *UAS*-GFP *UAS*-*scrib-IR*/+; *UAS*-*LacZ-IR*/+, **d** *ptc*-Gal4 *UAS*-GFP *UAS*-*scrib-IR*/+; *UAS*-*dGLYAT-IR*/+

To further confirm the effect of *dGLYAT* on cell invasion, we checked other markers of epithelial-mesenchymal transition (EMT), a critical process in malignant transformation [18]. During the process of EMT, expressions of cell-cell junction proteins, such as E-cadherin, are switched off in epithelial cells [19]. Consistently, loss-of-*scrib* resulted in reduced expression of E-cadherin, as the fluorescent signal of E-cadherin antibody is significantly weakened along the A/P compartment boundary in the wing pouch area (Fig. 2a, b). This phenotype was suppressed by expression of *dGLYAT-IR*, but not *LacZ-IR* (Fig. 2c, d). Taken together, these data suggest that *dGLYAT* is required for cell polarity disruption-induced EMT-like cell migration.

### ***dGLYAT*is necessary for Egr-JNK pathway-triggered invasive cell migration**

**Fig. 3:**
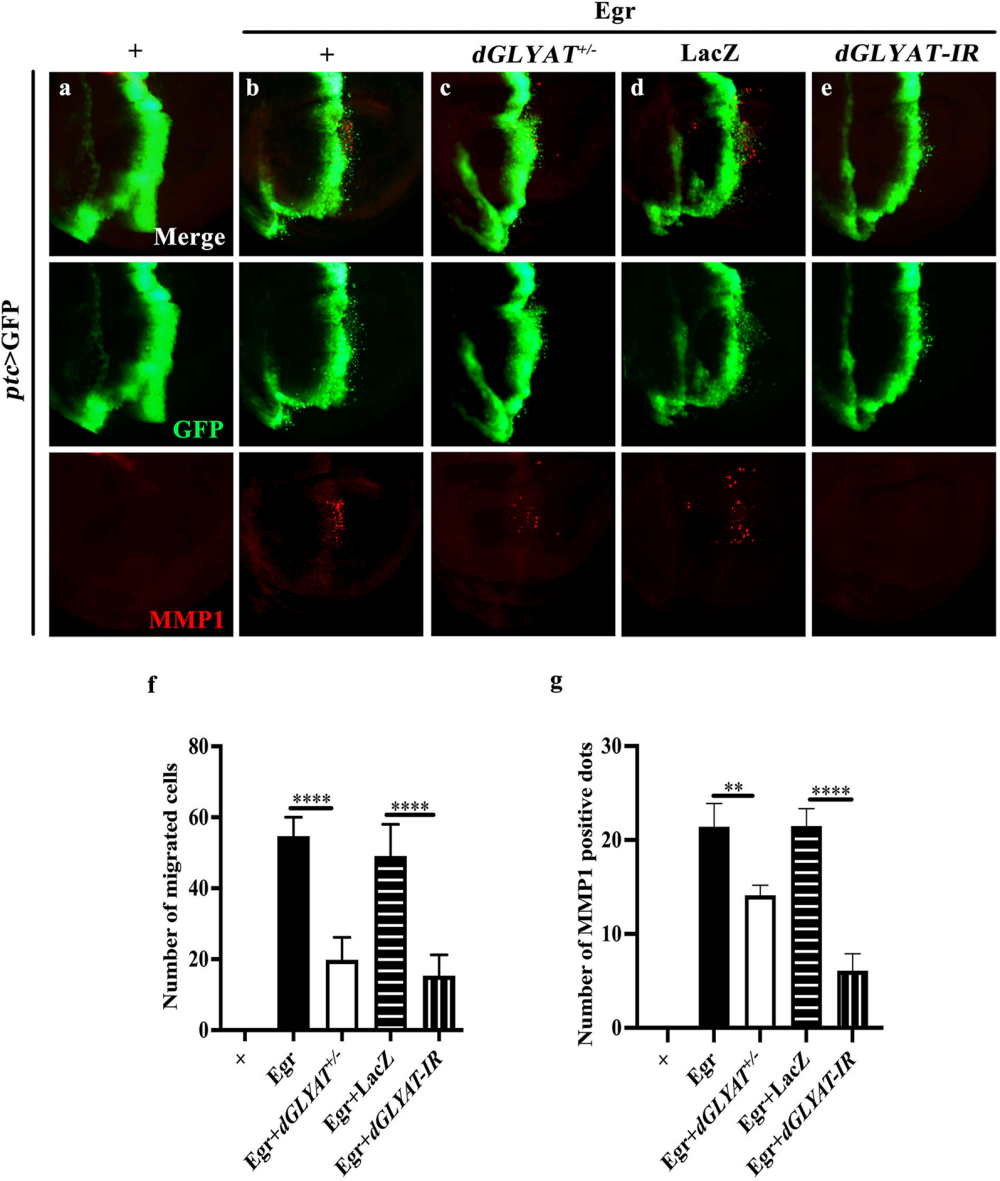
***dGLYAT*** is necessary for Eiger-JNK pathway-induced invasive cell migration. Fluorescent micrographs of third instar larval wing discs are shown (**a**-**e**). Compared with the *ptc* > GFP control (**a**), *ptc* > Egr-induced cell migration and up-regulated MMP1 expression (**b**) were suppressed by *dGLYAT* mutation (**c**) or RNAi (**e**), but not by LacZ expression (**d**). **f** Statistic of number of migrated cells is shown (left to right: n = 10, n = 12, n = 10. n = 12, n = 16). **g** Statistic of number of MMP1 positive dots is shown (left to right: n = 10, n = 22, n = 26. n = 27, n = 25). *t*-test was used to compute *P*-values. **P < 0.01, ****P < 0.0001. Detailed genotypes: **a** *ptc*-Gal4 *UAS*-GFP/+, **b** *ptc*-Gal4 *UAS*-GFP *UAS*-Egr ^*Regg1*^/+, **c** *ptc*-Gal4 *UAS*-GFP *UAS*-Egr^*Regg1*^/*dGLYAT*^*c02982*^, **d** *ptc*-Gal4 *UAS*-GFP *UAS*-Egr^*Regg1*^/+; *UAS*-LacZ/+, **e** *ptc*-Gal4 *UAS*-GFP *UAS*-Egr^*Regg1*^/+; *UAS*-*dGLYAT-IR*/+

*Scrib* depletion triggers Eiger-JNK pathway-mediated invasive cell migration [15]. To investigate whether *dGLYAT* is involved in Egr-JNK pathway-activated cell migration, we used *ptc*-Gal4 to drive ectopic Egr expression. Compared with the control (Fig. 3a), ectopic expression of Egr driven by *ptc*-Gal4 resulted in invasive cell migration, accompanied by up-regulated MMP1 expression (Fig. 3b). Both phenotypes were considerably suppressed in heterozygous *dGLYAT* mutants, or by expressing *dGLYAT-IR*, but not LacZ (Fig. 3c-e, quantified in Fig. 3f, g). As MMP1 also serves as a reporter of JNK signaling, these results suggest that *dGLYAT* is necessary for Egr-JNK pathway-triggered invasive cell migration.

### ***dGLYAT*** is required for cell polarity disruption-induced JNK pathway activation

**Fig. 4:**
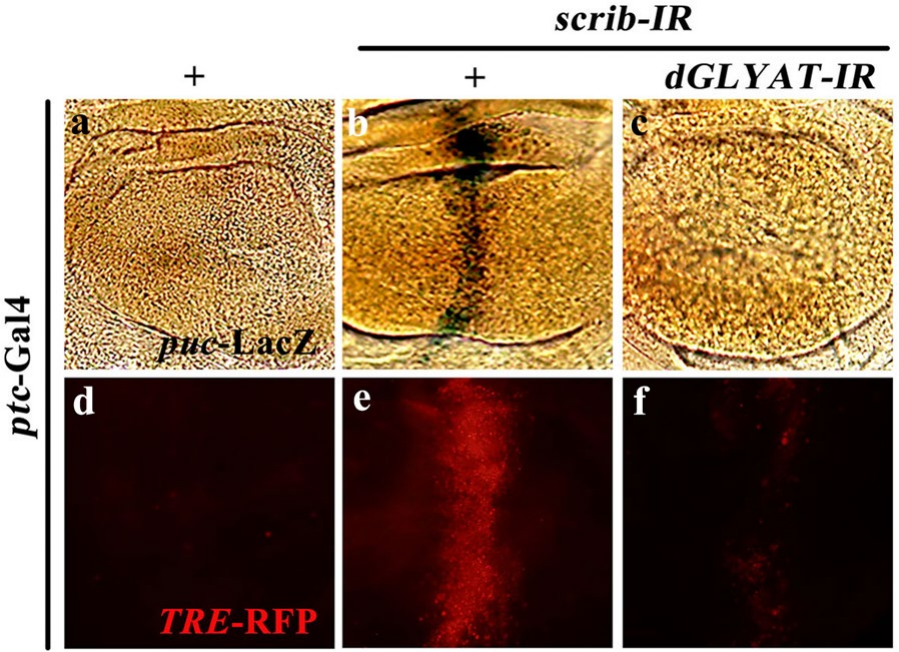
***dGLYAT*** is required for cell polarity disruption-induced JNK pathway activation. Light micrographs of third instar wing discs with X-gal staining (**a**-**c**) and fluorescent micrographs of third instar wing discs (**d**-**f**) are shown. Compared with the *ptc-*Gal4 control (**a**, **d**), *ptc* > *scrib-IR* activated *puc*-LacZ (**b**) and *TRE*-RFP (**e**) expression, which were suppressed by *dGLYAT* RNAi (**c**, **f**). Detailed genotypes: **a** *ptc*-Gal4 *UAS*-GFP/+; *puc*-LacZ/+, **b** *ptc*-Gal4 *UAS*-GFP *UAS*-*scrib-IR*/+; *puc*-LacZ/+, **c** *ptc*-Gal4 *UAS*-GFP *UAS*-*scrib-IR*/+; *puc-*LacZ/*UAS*-*dGLYAT-IR*, **d** *ptc*-Gal4 *UAS*-GFP/*TRE-*RFP, **e** *ptc*-Gal4 *UAS*-GFP *UAS-scrib-IR/TRE-*RFP, **f** *ptc*-Gal4 *UAS*-GFP *UAS-scrib-IR/TRE-*RFP; *UAS*-*dGLYAT-IR*/+

As loss-of-cell polarity-induced cell invasion depends on JNK signaling, and depletion of *dGLYAT* impedes *ptc > scrib-IR*-induced cell invasion and MMP1 expression, a transcriptional target of JNK signaling, we proposed that *dGLYAT* might be required for cell polarity disruption-triggered JNK pathway activation. To test this possibility, we examined the expression of two well-recognized JNK pathway reporters, *puc*-LacZ and *TRE*-RFP [20]. In agreement with previous reports, knockdown of *scrib* along the A/P compartment boundary triggered JNK pathway activation, as indicated by elevated expression of *puc*-LacZ (Fig. 4a, b) and *TRE*-RFP (Fig. 4d, e), both of which were significantly suppressed by knockdown of *dGLYAT* (Fig. 4c, f). Collectively, these results suggest that *dGLYAT* is required for loss-of-cell polarity-induced JNK pathway activation. ***dGLYAT*** **regulates** ***Gadd45*** **transcription**.

**Fig. 5:**
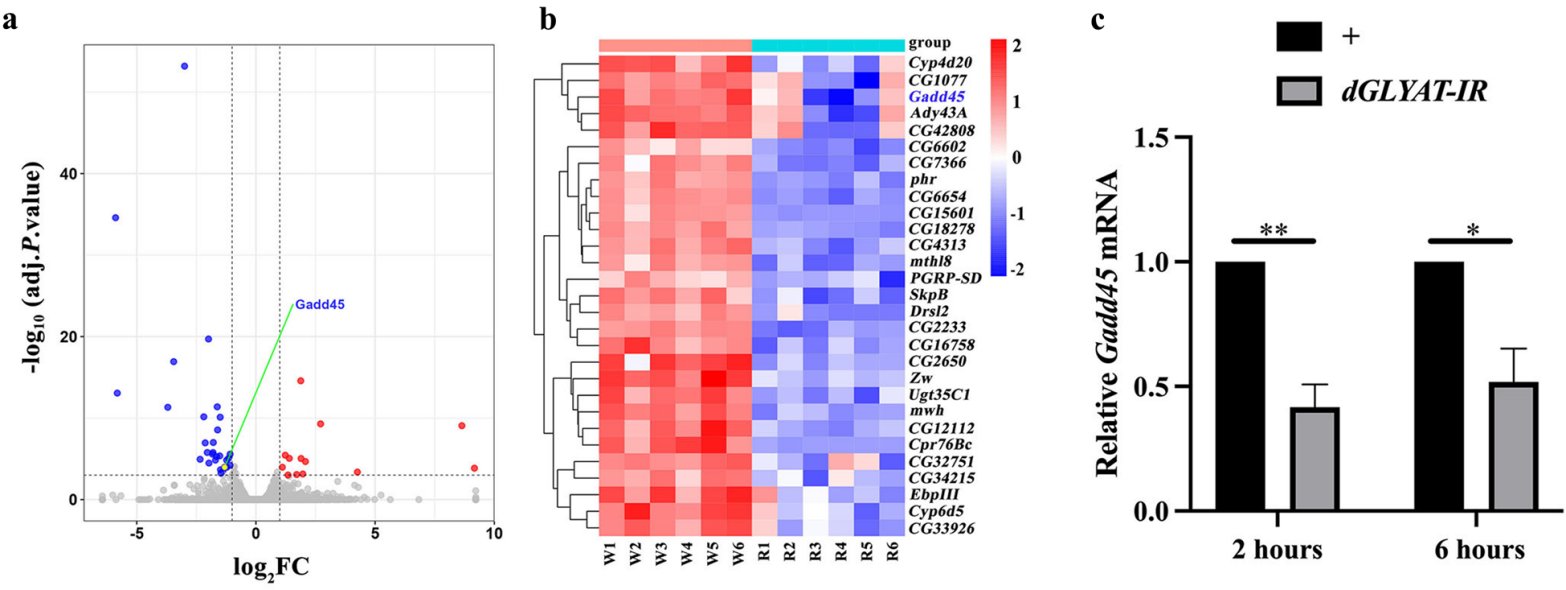
*Gadd45* mRNA level is reduced upon *dGLYAT* depletion. **a** Volcano plot between *dGLYAT* RNAi and control groups. Compared with the *hs*-Gal4 control groups, there are 29 down-regulated genes (indicated by blue points) and 13 up-regulated genes (showed by red points) in the *hs > dGLYAT-IR* groups, when log_2_FC > 1.0 or <-1.0 and adjusted *P* value < 0.001. Grey dotted lines show thresholds of log_2_FC (1.0 and − 1.0) and adjusted *P* value (0.001). **b** Heat map of 29 down-regulated genes between *dGLYAT* RNAi (R1-R6) and control (W1-W6) groups when log_2_FC<-1.0 and adjusted *P* value < 0.001. Genes showing significant changes are listed in Additional file 1: Table S1. **c** RT-qPCR data showed that *Gadd45* mRNA level was decreased upon *dGLYAT* depletion. *P < 0.05, **P < 0.01. Third instar larvae were heat shocked at 37℃ for 30 min and recovered at 29℃ for 2 h (**c**) or 6 h (**a**-**c**) prior to tissues dissection and RNA extraction

According to the Flybase (http://flybase.org/reports/FBgn0054010), *dGLYAT* encodes a Gcn5-related N-acetyltransferases (GNAT) family member that catalyzes the transfer of an acetyl moiety from Coenzyme A (Ac-CoA) to diverse substrates [21]. GNATs are evolutionarily conserved regulators that acetylate key amine of small molecules and proteins involved in numerous cellular processes. Some members of GNAT superfamily are involved in histone acetylation [22], which affects gene transcription [23]. Based on these information, we proposed that dGLYAT might be required for the transcriptional activation of JNK pathway positive regulator(s). In this scenario, loss of *dGLYAT* would compromise JNK pathway activation via reduced expression of the positive regulator(s). To find the potential regulator(s), we conducted the whole genome mRNA-seq assays, and analyzed differentially expressed genes (DEGs) between control and *dGLYAT*-depleted groups. We crossed *hs*-Gal4 with *w*^*1118*^ or *dGLYAT* RNAi lines, which serves as the control group or the experimental group, respectively. Third instar larvae were subjected to heat shock at 37 °C for 30 min, recovered at 29 °C for 6 h, and dissected for transcriptome sequencing. We found that 13 genes were up-regulated and 29 genes down-regulated upon *dGLYAT* knockdown (Fig. 5a, log_2_FC ≥ 1.0 or log_2_FC ≤ − 1.0, adjusted *P* value < 0.001 and Additional file 1: Table S1). We paid specific attention to the 29 down-regulated genes, whose expression profiles are presented (Fig. 5b). Intriguingly, among the 29 down-regulated genes, Gadd45 has been previously reported as a positive regulator of JNK pathway in apoptosis and egg asymmetric development [24, 25]. The mammalian GADD45 family consists of three members, GADD45a, GADD45b, and GADD45G. which interacts with cellular proteins in response to physiological or environmental stressors, and participates in cell cycle arrest, DNA repair, apoptosis, cell survival and senescence [26]. We performed reverse transcription-quantitative polymerase chain reaction (RT-qPCR) assay to validate the mRNA-seq results, and confirmed that *Gadd45* mRNA level was significantly reduced 2 and 6 h after heat shock-induced *dGLYAT* knockdown (Fig. 5c). The relative short time interval (2 h) between *dGLYAT* knockdown and *Gadd45* mRNA reduction implies dGLYAT may directly affect *Gadd45* transcription.

### Loss of ***Gadd45*** suppresses ***scrib*** depletion-induced JNK activation and cell invasion

**Fig. 6:**
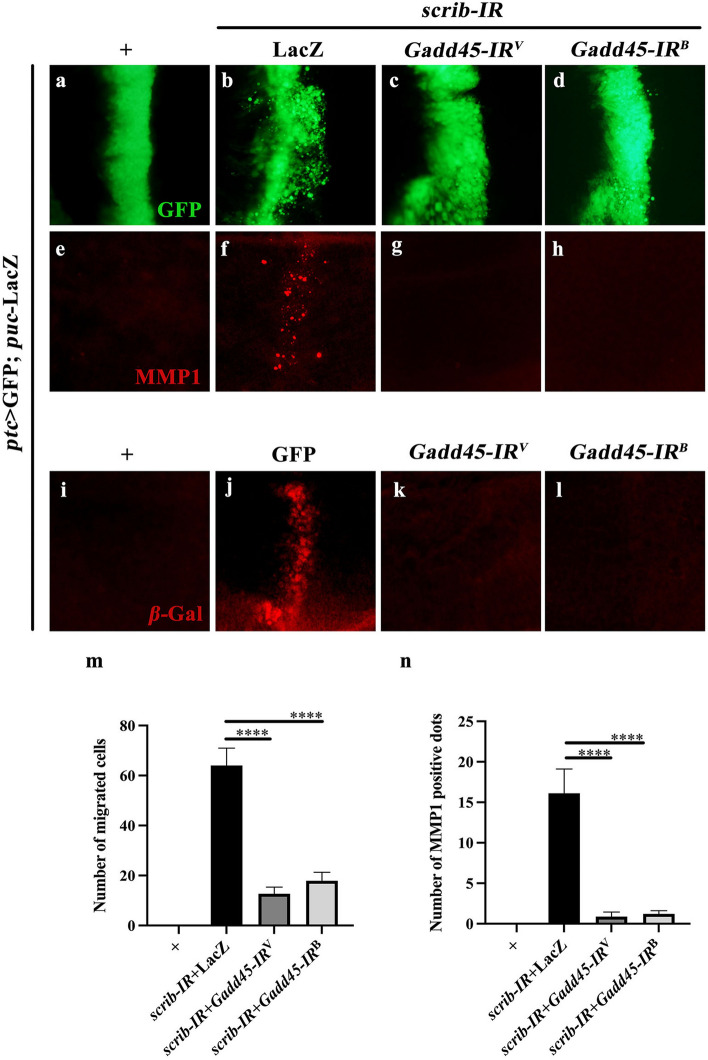
*Gadd45* knockdown suppresses *scrib* depletion-induced JNK activation and cell invasion. Compared with the *ptc*-Gal4 control (**a**, **e**, **i**), *scrib* RNAi-induced cell migration (**b**), elevated expression of MMP1 (**f**) and *puc*-LacZ (**j**) were significantly suppressed by expressing two independent *Gadd45* RNAi (**c**, **d**, **g**, **h** and **k**, **l**). **m** Statistic of number of migrated cells is shown (left to right: n = 10, n = 12, n = 9. n = 10). **n** Statistic of number of MMP1 positive dots is shown (left to right: n = 9, n = 9, n = 9, n = 9). One-way ANOVA was used to compute *P*-values, ****P < 0.0001. Larvae were reared at 25 °C. Detailed genotypes: **a**, **e**, **i** *ptc*-Gal4 *UAS*-GFP/+; *puc*-LacZ/+, **b**, **f** *ptc*-Gal4 *UAS*-GFP *UAS*-*scrib-IR*; *puc*-LacZ /*UAS*-LacZ, **c**, **g**, **k** *ptc*-Gal4 *UAS*-GFP *UAS*-*scrib-IR*/*UAS*-*Gadd45-IR*^*V100413*^; *puc*-LacZ/+, **d**, **h**, **l** *ptc*-Gal4 *UAS*-GFP *UAS*-*scrib-IR*/*UAS*-*Gadd45-IR*^*BL35023*^; *puc*-LacZ/+, **j** *ptc* > GFP *UAS*-*scrib-IR*; *puc*-LacZ/*UAS*-GFP

Given that loss of *dGLYAT* decreased *Gadd45* mRNA expression and suppressed JNK-dependent cell invasion, we hypothesized that dGLYAT regulates JNK-dependent cell invasion through Gadd45. In agreement with this assumption, *ptc* > *scrib-IR*-induced invasive cell migration was significantly suppressed by knockdown of *Gadd45* with two independent RNAi lines (Fig. 6a-d, quantified in Fig. 6 m). The knockdown efficiencies of *Gadd45* RNAi lines were verified by RT-qPCR analysis (Additional file 2: Figure S1). Furthermore, *scrib* depletion-induced MMP1 upregulation was impeded by knockdown of *Gadd45* (Fig. 6e-h, quantified in Fig. 6n). We also checked the expression of another JNK pathway reporter *puc*-LacZ, represented by anti-${\upbeta }$-Gal immunostaining. We found that *scrib* depletion-elevated *puc*-LacZ expression was blocked by loss of *Gadd45* (Fig. 6i-l).

JNK signaling also regulates other in vivo functions, in particular, cell death. For instance, overexpression of Egr driven by *GMR*-Gal4 (*GMR* > Egr) in the developing eyes triggers cell death in the larval eye discs, indicated by AO staining (Additional file 2: Figure S2g, h), and generates a small eye phenotype in the adults (Additional file 2: Figure S2a, b). Both phenotypes were significantly suppressed by knockdown of *dGLYAT* or *Gadd45*, while expression of a dominant-negative form of Basket (Bsk^DN^) served as a positive control (Additional file 2: Figure S2c–f, i–l, quantified in Additional file 2: Figure S2m, n). Collectively, these results suggest that dGLYAT and Gadd45 are broadly required for JNK signaling in vivo.

## Discussion

In this study we found that loss of *Drosophila GLYAT* suppresses cell polarity disruption-induced JNK-dependent cell invasion. Furthermore, loss of *dGLYAT* impedes Egr-JNK pathway-triggered invasive cell migration. Moreover, dGLYAT regulates the transcription of *Gadd45*, a positive regulator of JNK signaling in apoptosis and egg development. Finally, depletion of *Gadd45* blocks loss-of-cell polarity-triggered JNK activation and cell invasion. Thus, dGLYAT regulates Gadd45-mediated JNK activation and EMT-like cell migration in *Drosophila*.

Consistent with our fly data, in the UALCAN cancer database containing TCGA data, the expression of GLYATL1 and GADD45G are elevated in breast invasive carcinoma, compared with normal tissues (Additional file 2: Figure S3a, b). In addition, there is a positive correlation between *GLYATL1* and *GADD45G* mRNA expression in GEPIA database (Additional file 2: Figure S3d). More importantly, GEPIA confirmed that higher *GLYATL1* mRNA is associated with poor overall survival in breast cancer patients (Additional file 2: Figure S3c). These data imply that the role of dGLYAT and Gadd45 in cell invasion might be conserved by their human orthologs in breast cancer.

The role of GADD45 in P38 and JNK signaling have been extensively studied, yet divergent mechanisms were reported. GADD45 proteins activate MTK1 by promoting its dimerization and autophosphorylation [27], or bind and activate MTK1 MAPKKK to regulate P38 and JNK signaling upon environmental stress [28]. In addition, GADD45$\alpha$ could interact directly with P38 and regulates oncogene-induced growth [29], while GADD45$\beta$ interacts with ASK1 and MKK7 to regulate both JNK and P38 [30–32]. GADD45 proteins could execute either tumor suppressor or tumor promoter function in tumor initiation [33], yet their roles in tumor invasion have not been explored previously. The *Drosophila* genome encodes only one GADD45 member, Gadd45, which is involved in inflammatory response, egg development, wing disc regeneration and lifespan [24, 25, 34]. Gadd45 has been shown as a positive regulator of JNK signaling in germline development and cell death [24, 25], yet its role in JNK-mediated cell invasion has not been known previously. In this study, we found that Gadd45 is required for cell polarity disruption-induced JNK-mediated cell invasion in *Drosophila*, suggesting a potential role of GADD45 in tumor invasion and cancer progression, which deserves further investigation.

Although we found that dGLYAT regulates *Gadd45* mRNA expression, the underlying mechanism remains unknown. We hypothesize that dGLYAT, which contains a GNAT domain, may promote histone acetylation at the promoter of *Gadd45*. In support of this assumption, the yeast GNAT domain-containing protein Gcn5 (general control nonderepressible-5) is reported to regulate *PHO5* transcription by promoting histone acetylation at its promoter [35], and this histone acetylation activity has been conserved by Gcn5 homologs in *Tetrahymena* and human [36]. Future studies are needed to address whether GLYAT family proteins promote histone acetylation, and whether this activity is required for their roles in cancer initiation and progression.

## Conclusions

In summary, we found dGLYAT is required for JNK-mediated cell invasion. Through analyzing mRNA-seq results, we found *Gadd45* mRNA expression is reduced upon *dGLYAT* depletion. Downregulation of *Gadd45* also suppressed JNK-mediated cell invasion and cell death.

## Methods

### ***Drosophila*** genetics and stocks

Fly stocks were raised on standard cornmeal and agar medium at 25 °C. For cell migration assay, larvae were reared at 29 °C unless indicated. Fly strains used in this study are as follow: *w*^*1118*^, *ptc*-Gal4, *UAS*-GFP, *UAS*-*scrib-RNAi*, *puc*^*E69*^, *TRE*-RFP, *UAS-*Egr^Regg1^, *dGLYAT*^*c02982*^ and *UAS*-*dGLYAT*-*IR* have been described previously [14]. *UAS-Gadd45-RNAi* (V100413 and BL35023) were gifts from Dr. Erjun Ling (Chinese Academy of Sciences, China).

### Immunohistochemistry

Antibody staining was performed according to standard procedures. The following primary antibodies were used: mouse anti-MMP1 (3A6B4, 1:200, Developmental Studies Hybridoma Bank, DSHB), Rat anti-E-cad (DCAD2-c, 1:100, Developmental Studies Hybridoma Bank, DSHB) and mouse anti $\beta$-gal (40-1a, 1:500, Developmental Studies Hybridoma Bank, DSHB). The following secondary antibodies were used: anti-mouse CY3 (A11032, 1:1000, Cell Signaling Technology) and anti-Rat CY3 (104,086, 1:500, Jackson Immuno research).

### X-gal staining

Wing discs were dissected from third instar larvae in PBST (1× PBS pH 7.0, 0.1% Triton X-100) and stained for β-galactosidase activity as described [37].

### RNA library preparation and data analysis

Third instar larvae were subjected to heat shock at 37 °C for 30 min and recovered at 29 °C for 6 h prior to tissues dissection. RNA extraction, library construction and sequencing were performed by Hua Gen Biotechnology (Shanghai, China). The sequencing platform is Illumina. The quality of sequenced raw reads was controlled by the FastQ Screen program. The reference genome sequence is *Drosophila melanogaster (BDGP6.28)* downloaded from Ensembl. (http://www.ensembl.org/info/data/ftp/index.html). Differentially expressed genes (DEGs) were identified by comparing the expression levels of genes between *hs* > *dGLYAT* RNAi and *hs*-Gal4 control groups. The threshold for DEGs was set at *P-* adjusted < 0.001 and log_2_ fold change (log_2_FC) ≥ 1.0 or ≤ − 1.0.

### RT-qPCR

Total RNAs were extracted from fifteen third instar larval tissues of indicated genotypes with Trizol (Ambion, Life Technologies, Carlsbad, CA, USA) following the protocol of RNA preparation kit, and quantitative polymerase chain reaction (qPCR) was performed using SYBR Green PCR Premix Kit (TaKaRa). The primers used are as follow:

**Table:** 

Gene	Forward primer	Reverse primer
*rp49*	TCTCCTTGCGCTTCTTGGA	TACAGGCCCAAGATCGTGAA
*dGLYAT*	ATACCATTAAGGGAACCCCAGA	TGACCCAAATTCAGCCAATATGC
*Gadd45*	ATCGGACGCACCATCAAGTC	TGTCGTTCTCGTAGCAAAAGG

### UALCAN and GEPIA cancer databases

The UALCAN cancer database (http://ualcan.path.uab.edu/analysis.html) is a comprehensive web source that provide the data from The Cancer Genome Atlas (TCGA) [38]. The GEPIA database (http://gepia2.cancer-pku.cn/#index) contains data from TCGA and Genotype-Tissue Expression (GTEx) project [39].

## Supplementary Information

**Additional file 1: Table S1**. mRNA-seq data between control and dGLYAT-depleted groups.

**Additional file 2: Figure S1**. The knock-down efficiencies of RNAi lines. The efficiencies of dGLYAT and GADD45 RNAi lines were measured by RT-qPCR. dGLYAT mRNA and Gadd45 mRNA levels were significantly downregulated by the expression of their corresponding RNAi. Third instar larvae were subjected to heat shock at 37°C for 30 minutes in the water bath and recovered for 2 hours at 29°C. Larval discs were dissected for RT-qPCR. Error bars represents standard deviation from three independent experiments. One-way ANOVA test was used to compute P-values, ****P<0.0001.** Figure S2**. Depletion of dGLYAT or Gadd45 suppresses GMR>Egr-induced cell death. Light micrographs of Drosophila adult eyes (a–f) and fluorescent micrographs of third instar larval eye discs (g–l) are shown. Compared with the GMR-Gal4 controls (a, g), GMR>Egr induces a small eye phenotype in adults (b) and massive cell death in third instar larval eye discs with AO staining (h). Both phenotypes were suppressed by knockdown of dGLYAT or Gadd45 (c-e and i-k). BskDN serves as a positive control (f, l). (m) Statistic of eyes size is shown (from left to right: n =7, n=10, n=10, n=10, n=9, n=5). (n) Statistic of AO-positive cell number is shown (from left to right: n =10, n=11, n=13, n=12, n=7, n=10), One-way ANOVA test was used to compute P-values, ****P<0.0001.** Figure S3**. Characterization of GLYATL1 and GADD45G in breast cancer. (a, b) Transcriptome sequencing of breast cancer. Expression of GLYATL1 and GADD45G in normal and tumor tissues were measured in transcript per million utilizing the TCGA data set. The tumor tissues show higher expression of GLYATL1 and GADD45G than normal tissues. (c) Survival analysis of GLYATL1 in breast cancer patients. The survival of breast cancer patients with higher GLYATL1 expression was significantly worse (P<0.05). (d) The expression relationship between GLYATL1 and GADD45G in breast cancer using GEPIA database: a positive correlation between expression of GLYATL1 and GADD45G (P=7.5e-08, R=0.15).

## Data Availability

The data that support the finding of this study are available from the corresponding author upon reasonable request.

## Abbreviations

Csk: C-terminal SRC kinase
JNK: c-Jun N-terminal kinase
GLYAT: Glycine N-acyltransferases
Gadd45: Growth Arrest and DNA Damage-inducible 45
MMP1: Matrix metalloprotease 1
EMT: Epithelial-mesenchymal transition
GNAT: Gcn5-related N-acetyltransferases
Ac-CoA: Acetyl moiety from Coenzyme A
DEGs: Differentially expressed genes
RT-qPCR: Reverse transcription-quantitative polymerase chain reaction
TCGA: The Cancer Genome Atlas

## References

[1] Welch DR, Hurst DR (2019). Defining the hallmarks of metastasis. Cancer Res.

[2] Stoletov K, Beatty PH, Lewis JD (2020). Novel therapeutic targets for cancer metastasis. Expert Rev Anticancer Ther.

[3] Miles WO, Dyson NJ, Walker JA (2011). Modeling tumor invasion and metastasis in Drosophila. Dis Model Mech.

[4] Vidal M, Larson DE, Cagan RL (2006). Csk-deficient boundary cells are eliminated from normal Drosophila epithelia by exclusion, migration, and apoptosis. Dev Cell.

[5] Shizu R, Min J, Sobhany M, Pedersen LC, Mutoh S, Negishi M (2018). Interaction of the phosphorylated DNA-binding domain in nuclear receptor CAR with its ligand-binding domain regulates CAR activation. J Biol Chem.

[6] Takatsu Y, Nakamura M, Stapleton M, Danos MC, Matsumoto K, O’Connor MB (2000). TAK1 participates in c-Jun N-terminal kinase signaling during Drosophila development. Mol Cell Biol.

[7] Glise B, Bourbon H, Noselli S (1995). hemipterous encodes a novel Drosophila MAP kinase kinase, required for epithelial cell sheet movement. Cell.

[8] Chen HW, Marinissen MJ, Oh SW, Chen X, Melnick M, Perrimon N (2002). CKA, a novel multidomain protein, regulates the JUN N-terminal kinase signal transduction pathway in Drosophila. Mol Cell Biol.

[9] Perkins KK, Dailey GM, Tjian R (1988). Novel Jun- and Fos-related proteins in Drosophila are functionally homologous to enhancer factor AP-1. The EMBO journal.

[10] Martin-Blanco E, Gampel A, Ring J, Virdee K, Kirov N, Tolkovsky AM (1998). puckered encodes a phosphatase that mediates a feedback loop regulating JNK activity during dorsal closure in Drosophila. Genes Dev.

[11] La Marca JE, Richardson HE, Two-Faced (2020). Roles of JNK signalling during tumourigenesis in the Drosophila model. Front Cell Dev Biol.

[12] Matsuo M, Terai K, Kameda N, Matsumoto A, Kurokawa Y, Funase Y (2012). Designation of enzyme activity of glycine-N-acyltransferase family genes and depression of glycine-N-acyltransferase in human hepatocellular carcinoma. Biochem Biophys Res Commun.

[13] Guan R, Hong W, Huang J, Peng T, Zhao Z, Lin Y (2020). The expression and prognostic value of GLYATL1 and its potential role in hepatocellular carcinoma. J Gastrointest Oncol.

[14] Ren P, Li W, Xue L (2017). GLYAT regulates JNK-mediated cell death in Drosophila. Sci Rep.

[15] Wu C, Ding X, Li Z, Huang Y, Xu Q, Zou R (2021). CtBP modulates Snail-mediated tumor invasion in Drosophila. Cell Death Discov.

[16] Zhang S, Guo X, Wu H, Sun Y, Ma X, Li J (2019). Wingless modulates activator protein-1-mediated tumor invasion. Oncogene.

[17] Sun Y, Zhang D, Guo X, Li W, Li C, Luo J (2019). MKK3 modulates JNK-dependent cell migration and invasion. Cell Death Dis.

[18] Thiery JP (2002). Epithelial-mesenchymal transitions in tumour progression. Nat Rev Cancer.

[19] Lamouille S, Xu J, Derynck R (2014). Molecular mechanisms of epithelial-mesenchymal transition. Nat Rev Mol Cell Biol.

[20] Poulton JS, Cuningham JC, Peifer M (2014). Acentrosomal Drosophila epithelial cells exhibit abnormal cell division, leading to cell death and compensatory proliferation. Dev Cell.

[21] Salah Ud-Din AI, Tikhomirova A, Roujeinikova A (2016). Structure and functional diversity of GCN5-related N-acetyltransferases (GNAT). Int J Mol Sci..

[22] Vetting MW, LP SdC, Yu M, Hegde SS, Magnet S, Roderick SL (2005). Structure and functions of the GNAT superfamily of acetyltransferases. Arch Biochem Biophys.

[23] Mizzen CA, Allis CD (1998). Linking histone acetylation to transcriptional regulation. Cell Mol Life Sci CMLS.

[24] Camilleri-Robles C, Serras F, Corominas M (2019). Role of D-GADD45 in JNK-dependent apoptosis and regeneration in Drosophila. Genes..

[25] Peretz G, Bakhrat A, Abdu U (2007). Expression of the Drosophila melanogaster GADD45 homolog (CG11086) affects egg asymmetric development that is mediated by the c-Jun N-terminal kinase pathway. Genetics.

[26] Salvador JM, Brown-Clay JD, Fornace AJ (2013). Jr. Gadd45 in stress signaling, cell cycle control, and apoptosis. Adv Exp Med Biol.

[27] Miyake Z, Takekawa M, Ge Q, Saito H (2007). Activation of MTK1/MEKK4 by GADD45 through induced N-C dissociation and dimerization-mediated trans autophosphorylation of the MTK1 kinase domain. Mol Cell Biol.

[28] Takekawa M, Saito H (1998). A family of stress-inducible GADD45-like proteins mediate activation of the stress-responsive MTK1/MEKK4 MAPKKK. Cell.

[29] Bulavin DV, Kovalsky O, Hollander MC, Fornace AJ (2003). Jr. Loss of oncogenic H-ras-induced cell cycle arrest and p38 mitogen-activated protein kinase activation by disruption of Gadd45a. Mol Cell Biol.

[30] Papa S, Zazzeroni F, Bubici C, Jayawardena S, Alvarez K, Matsuda S (2004). Gadd45 beta mediates the NF-kappa B suppression of JNK signalling by targeting MKK7/JNKK2. Nat Cell Biol.

[31] Papa S, Monti SM, Vitale RM, Bubici C, Jayawardena S, Alvarez K (2007). Insights into the structural basis of the GADD45beta-mediated inactivation of the JNK kinase, MKK7/JNKK2. J Biol Chem.

[32] Ueda T, Kohama Y, Kuge A, Kido E, Sakurai H (2017). GADD45 family proteins suppress JNK signaling by targeting MKK7. Arch Biochem Biophys.

[33] Zhang L, Yang Z, Liu Y (2014). GADD45 proteins: roles in cellular senescence and tumor development. Exp Biol Med (Maywood).

[34] Plyusnina EN, Shaposhnikov MV, Moskalev AA (2011). Increase of Drosophila melanogaster lifespan due to D-GADD45 overexpression in the nervous system. Biogerontology.

[35] Gregory PD, Schmid A, Zavari M, Lui L, Berger SL, Horz W (1998). Absence of Gcn5 HAT activity defines a novel state in the opening of chromatin at the PHO5 promoter in yeast. Mol Cell.

[36] Candau R, Moore PA, Wang L, Barlev N, Ying CY, Rosen CA (1996). Identification of human proteins functionally conserved with the yeast putative adaptors ADA2 and GCN5. Mol Cell Biol.

[37] Wu C, Li Z, Ding X, Guo X, Sun Y, Wang X (2019). Snail modulates JNK-mediated cell death in Drosophila. Cell Death Dis.

[38] Chandrashekar DS, Bashel B, Balasubramanya SAH, Creighton CJ, Ponce-Rodriguez I, Chakravarthi B (2017). UALCAN: a portal for facilitating tumor subgroup gene expression and survival analyses. Neoplasia.

[39] Tang Z, Li C, Kang B, Gao G, Li C, Zhang Z (2017). GEPIA: a web server for cancer and normal gene expression profiling and interactive analyses. Nucleic Acids Res.

