# Supplementary material for: dGLYAT modulates Gadd45-mediated JNK activation and cell invasion
Source: Cell Div. 2022 Aug 6;17:4. doi: 10.1186/s13008-022-00080-5 (PMC9357319; doi:10.1186/s13008-022-00080-5)
Supplement: Supplementary file 2 — Additional file 2: Figure S1. The knock-down efficiencies of RNAi lines. The efficiencies of dGLYAT and GADD45 RNAi lines were measured by RT-qPCR. dGLYAT mRNA and Gadd45 mRNA levels were significantly downregulated by the expression of their corresponding RNAi. Third instar larvae were subjected to heat shock at 37°C for 30 minutes in the water bath and recovered for 2 hours at 29°C. Larval discs were dissected for RT-qPCR. Error bars represents standard deviation from three independent experiments. One-way ANOVA test was used to compute P-values, ****P<0.0001. Figure S2. Depletion of dGLYAT or Gadd45 suppresses GMR>Egr-induced cell death. Light micrographs of Drosophila adult eyes (a–f) and fluorescent micrographs of third instar larval eye discs (g–l) are shown. Compared with the GMR-Gal4 controls (a, g), GMR>Egr induces a small eye phenotype in adults (b) and massive cell death in third instar larval eye discs with AO staining (h). Both phenotypes were suppressed by knockdown of dGLYAT or Gadd45 (c-e and i-k). BskDN serves as a positive control (f, l). (m) Statistic of eyes size is shown (from left to right: n =7, n=10, n=10, n=10, n=9, n=5). (n) Statistic of AO-positive cell number is shown (from left to right: n =10, n=11, n=13, n=12, n=7, n=10), One-way ANOVA test was used to compute P-values, ****P<0.0001. Figure S3. Characterization of GLYATL1 and GADD45G in breast cancer. (a, b) Transcriptome sequencing of breast cancer. Expression of GLYATL1 and GADD45G in normal and tumor tissues were measured in transcript per million utilizing the TCGA data set. The tumor tissues show higher expression of GLYATL1 and GADD45G than normal tissues. (c) Survival analysis of GLYATL1 in breast cancer patients. The survival of breast cancer patients with higher GLYATL1 expression was significantly worse (P<0.05). (d) The expression relationship between GLYATL1 and GADD45G in breast cancer using GEPIA database: a positive correlation between expression of GLYATL1 and GADD45G ( [file 13008_2022_80_MOESM2_ESM.docx]

**dGLYAT Modulates Gadd45-Mediated JNK Activation and Cell Invasion**

Meng Xu^1^, Pu Ren^1, *^, Juhui Tian^1^, Lisha Xiao^1^, Ping Hu^1^, Ping Chen^1^, Wenzhe Li^1, *^ and Lei Xue^1, 2, *^

^1^ The First Rehabilitation Hospital of Shanghai, Shanghai Key Laboratory of Signaling and Diseases Research, School of Life Science and Technology, Tongji University, Shanghai, China.

^2^ Zhuhai Precision Medical Center, Guangdong Provincial Key Laboratory of Tumor Interventional Diagnosis and Treatment, Zhuhai People's Hospital, Zhuhai Hospital Affiliated with Jinan University, Zhuhai, Guangdong, China.

^*^ Correspondence: [lei.xue@tongji.edu.cn](mailto:lei.xue@tongji.edu.cn), [ren_pu123@qq.com](mailto:ren_pu123@qq.com), [lwz@tongji.edu.cn](mailto:lwz@tongji.edu.cn)

**Supplement Figure 1**


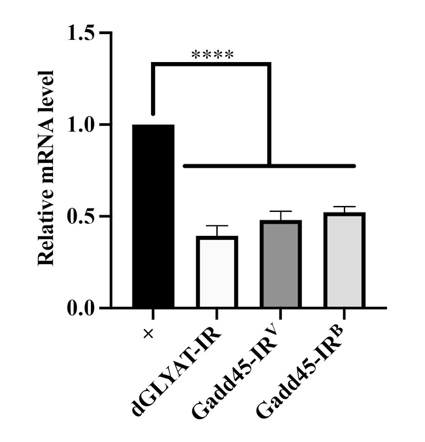


**Figure S1. The knock-down efficiencies of RNAi lines.** The efficiencies of *dGLYAT* and *GADD45* RNAi lines were measured by RT-qPCR. *dGLYAT* mRNA and *Gadd45* mRNA levels were significantly downregulated by the expression of their corresponding RNAi. Third instar larvae were subjected to heat shock at 37°C for 30 minutes in the water bath and recovered for 2 hours at 29°C. Larval discs were dissected for RT-qPCR. Error bars represents standard deviation from three independent experiments. One-way ANOVA test was used to compute *P*-values, *****P*<0.0001.

**Supplement Figure 2**


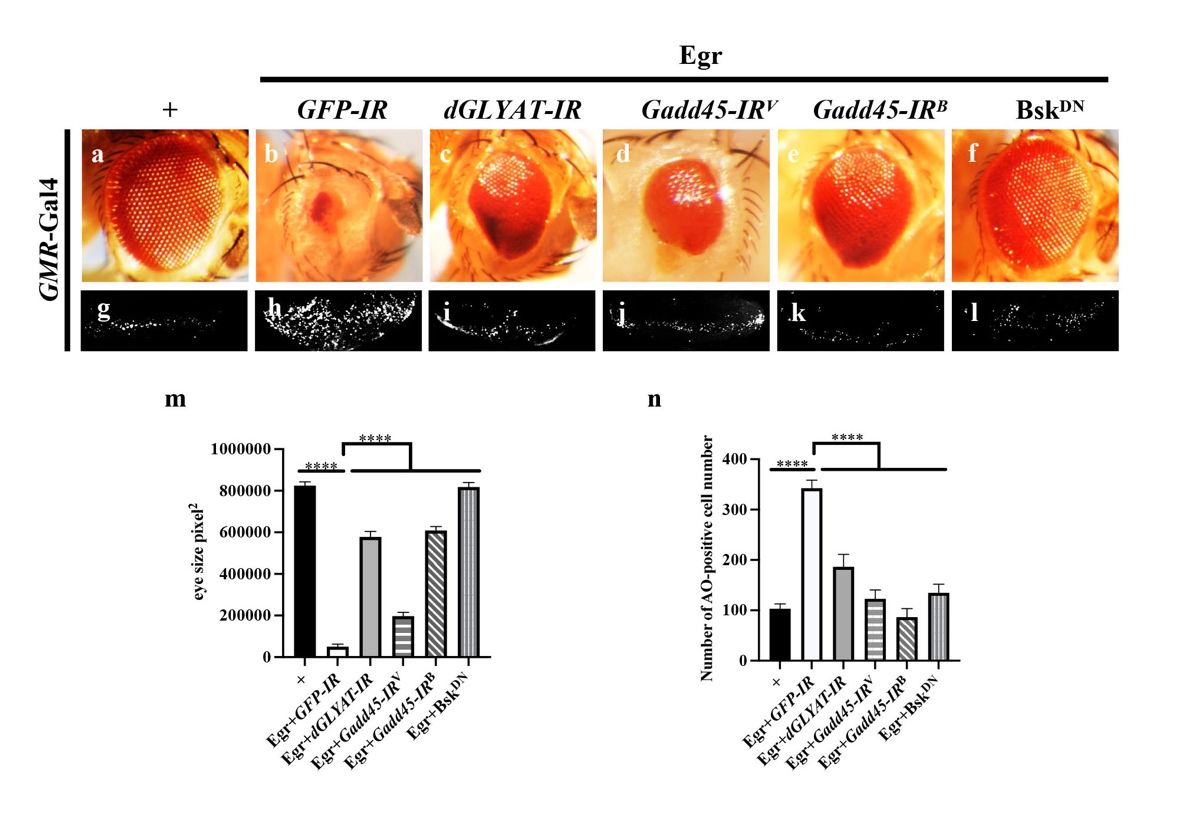


**Figure S2. Depletion of *dGLYAT* or *Gadd45* suppresses *GMR*>Egr-induced cell death.** Light micrographs of *Drosophila* adult eyes (a–f) and fluorescent micrographs of third instar larval eye discs (g–l) are shown. Compared with the *GMR*-Gal4 controls (a, g), *GMR*>Egr induces a small eye phenotype in adults (b) and massive cell death in third instar larval eye discs with AO staining (h). Both phenotypes were suppressed by knockdown of *dGLYAT* or *Gadd45* (c-e and i-k). Bsk^DN^ serves as a positive control (f, l). (m) Statistic of eyes size is shown (from left to right: n =7, n=10, n=10, n=10, n=9, n=5). (n) Statistic of AO-positive cell number is shown (from left to right: n =10, n=11, n=13, n=12, n=7, n=10), One-way ANOVA test was used to compute *P*-values, *****P*<0.0001.

**Supplement Figure 3**


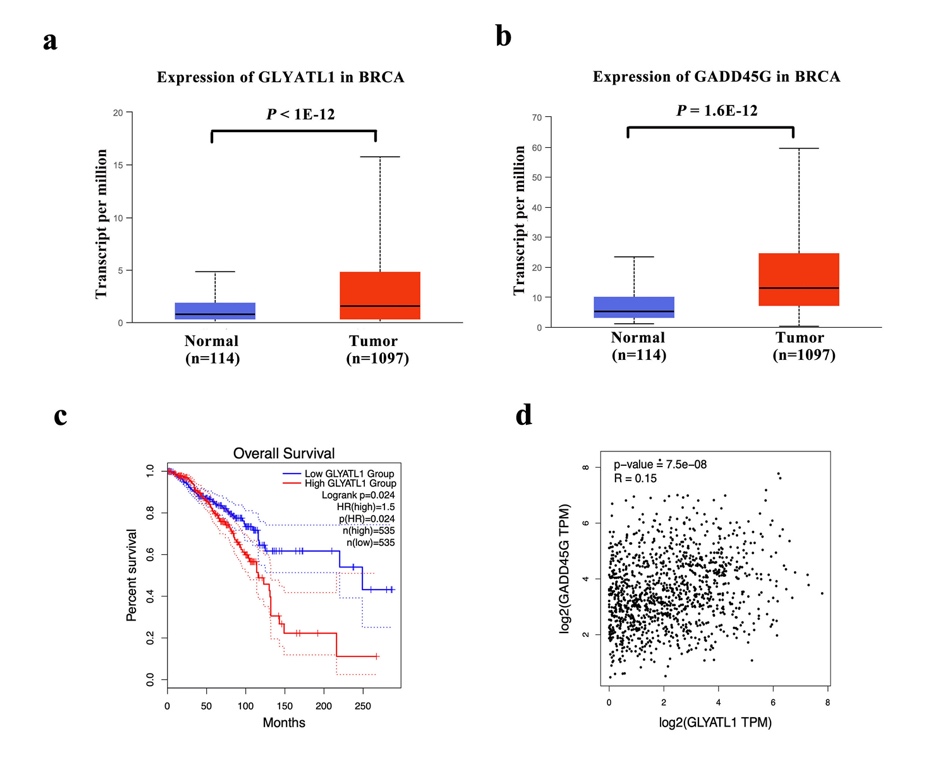


**Figure S3. Characterization of *GLYATL1* and *GADD45G* in breast cancer****.** (a, b) Transcriptome sequencing of breast cancer. Expression of *GLYATL1* and *GADD45G* in normal and tumor tissues were measured in transcript per million utilizing the TCGA data set. The tumor tissues show higher expression of *GLYATL1* and *GADD45G* than normal tissues. (c) Survival analysis of *GLYATL1* in breast cancer patients. The survival of breast cancer patients with higher *GLYATL1* expression was significantly worse (*P*<0.05). (d) The expression relationship between *GLYATL1* and *GADD45G* in breast cancer using GEPIA database: a positive correlation between expression of *GLYATL1* and *GADD45G* (*P*=7.5e-08, R=0.15).
